# The potential spread of severe footrot in Norway if no elimination programme had been initiated: a simulation model

**DOI:** 10.1186/s13567-015-0150-y

**Published:** 2015-02-20

**Authors:** Gry M Grøneng, Synnøve Vatn, Anja Bråthen Kristoffersen, Ola Nafstad, Petter Hopp

**Affiliations:** Norwegian Veterinary Institute, P.O. Box 750 Sentrum, NO-0106 Oslo, Norway; Animalia - Norwegian Meat and Poultry Research Centre, P.O. Box 396 Løren, NO-0513 Oslo, Norway

## Abstract

When severe footrot was detected in Norway in 2008, a surveillance programme was initiated and followed by an elimination programme. By 2013 the disease had spread to two of 19 counties and a total of 119 (1%) sheep flocks had been diagnosed with severe footrot. A simulation model was developed to estimate the potential spread of severe footrot in Norway and to estimate the relative importance of the different spreading routes. The model parameters were based on the rate of spread of the first 38 diagnosed cases and the management and climatic factors particular for Norway. The model showed that by 2013, severe footrot would have spread to six counties and infected 16% of the sheep flocks if no elimination programme had been initiated. If this is compared with the 1% of flocks that were diagnosed in Norway by 2013, there seems to be a large effect of the implemented footrot elimination programme. By 2035, it was estimated that severe footrot would have spread to 16 counties and 64% of the sheep flocks. Such an extensive spread would probably impose a large negative impact on the sheep industry and welfare of the sheep. The most effective way to curb the spread of severe footrot was by decreasing the within county infection rate. This could be achieved by decreasing the contact between flocks or by decreasing the environmental load of *D. nodosus*, for example by footbathing sheep, culling diseased sheep or eliminating severe footrot in the flock.

## Introduction

Footrot is well known in sheep-producing countries worldwide. The clinical signs range from mild inflammation of the interdigital skin to under-running and separation of the hoof horn from the sensitive tissues [1]. Footrot is a painful disease and causes lameness, poor welfare and affects ewe and lamb productivity [2,3]. *Dichelobacter nodosus* (*D. nodosus*), a Gram negative anaerobic bacterium, is the causative agent of footrot in small ruminants [1]. *D. nodosus* is divided into benign and virulent strains that can be differentiated in the laboratory by a gelatin gel test [4]. Clinical signs are often more severe when sheep are infected with the virulent *D. nodosus* strain than with the benign strain.

Severe footrot is a notifiable disease in Norway, and in 2008 the disease was diagnosed in the county of Rogaland in the south west of Norway [5]. This was the first detection of the disease in Norway since 1948 [6]. The term severe footrot has been used in Norway to include both flocks with diagnosed virulent strains of *D. nodosus* and flocks with severe clinical signs of footrot together with a positive PCR result but no bacterial isolates. A regional surveillance programme was initiated by the sheep industry in 2008, and in 2009, this was followed by a co-operative national elimination programme called the Healthy Feet Project (Animalia - Norwegian Meat and Poultry Research Centre). Between 2008 and 2011, clinical inspections were made of sheep in > 4500 sheep flocks. This includes close to 100% of the flocks in Rogaland. In addition, many of these flocks were inspected twice and some three or four times. By 2008, the disease had been detected in 1.5% of the flocks in Rogaland. The disease spread particularly rapidly in the municipality of Rennesøy, where 11.2% of the flocks were diagnosed with severe footrot in 2008 (Table 1). By 2012, severe footrot had only been detected in the county of Rogaland [7], but in 2013, virulent *D. nodosus* was diagnosed in 14 flocks in the county of Aust-Agder, also situated in the southern part of Norway. By the end of 2013, 119 flocks in Norway had been diagnosed with severe footrot. Of these, 118 are now declared to be free from footrot, and measures have been implemented to eliminate severe footrot from the remainder [8]. Epidemiological and bacteriological investigations have indicated that virulent *D. nodosus* was introduced into a single flock in the county of Rogaland in 2005 through the purchase of sheep from abroad and thereafter spread locally [9,10].

**Table 1:** **Data used for estimating minimum, mode and maximum infection rate of severe footrot in Rogaland**

	**Rogaland excluding Rennesøy (minimum)**		**Whole of Rogaland (mode)**		**Rennesøy (maximum)**	
**Number of sheep flocks**	2490		2597		107	
**Year**	Assumed infected	Predicted infected	Assumed infected	Predicted infected	Assumed infected	Predicted infected
**2005**	1	1	1	1	0	0
**2006**	ND	3	ND	3	1	1
**2007**	ND	9	ND	14	ND	4
**2008**	26	26	38	48	12	13
**Regional percentage of infected flocks in 2008**	1.0%		1.5%		11.2%	
**Estimated infection rate (β)**		**1.13**		**1.31**		**1.36**

ND = No data.

The estimates was based on data on the total number of sheep flocks and number of flocks assumed to be infected with severe footrot in the regions, from the introduction of the disease (2005 in Rogaland and 2006 in Rennesøy) until the initiation of the elimination programme in 2009. The predicted number of infected flocks in each region was the median value, of 2000 replicates of the model based on Equations 1–3. The infection rate is calculated using a constant yearly recovery rate of 5.3% and reversion rate of 1/3 of the infection rate.

The aim of this study was to estimate the potential spread of severe footrot in Norway if no elimination programme was implemented and estimate the importance of the different spreading routes of virulent *D. nodosus*. The developed model was based on the infection rate of the first diagnosed cases and the management and climatic factors specific to Norway.

## Material and methods

A stochastic compartmental model can be used to simulate spread of disease within a population [11]. In a SILI-compartmental model, the susceptible(*S*), infected(*I*) and low susceptible(*L*) compartments and the transmission of flocks between these compartments describe the infection dynamics of the population. The susceptible flocks are not, and have not been, infected with the agent causing the disease. The infected flocks have at least one sheep infected with the agent causing disease and could infect susceptible or low susceptible flocks. The low susceptible flocks do not have any animals carrying the infection*,* and have a smaller contact network than the susceptible flocks, hence are less at risk of acquiring a disease than the susceptible flocks. The low susceptible flocks comprise of flocks with natural barriers towards other sheep flocks (called isolated flocks) and flocks that have recovered from the disease and by this increased their biosecurity measures (called recovered flocks). The latent period is assumed to be zero, and the immunity period for a flock is negligible.

In the model, flocks are transferred from one compartment to another at different rates. The infection rate (β) is the rate at which susceptible flocks become infected. This is dependent on the number of contacts, and the risk of transmission of disease per contact. The recovery rate (σ) is the rate of recovery of infected flocks, and which are accordingly assigned to the low susceptible compartment. The reversion rate (Ɣ) is the rate at which low susceptible flocks become infected, and by this transferred to the infected compartment.

### Spread within subpopulations

Subpopulations can be defined if the spread of disease is not uniform in the population [12], but highly reduced from one geographical area to another. Each subpopulation is then modelled with their own SILI-compartmental model with their own infection, recovery and reversion rate. These rates are based on specific values for each subpopulation that influence the spread of the disease in question.

### Spread between subpopulations

Spread of disease is expected to be faster within the subpopulations than between two subpopulations as flocks within each subpopulation are expected to have more contact than flocks from two different subpopulations. Different types of contact between flocks in separate subpopulations may occur, leading to different transmission routes. Each transmission route between subpopulations are specified and quantified separately. Only susceptible flocks in the subpopulations are expected to be infected by other subpopulations as most of the low risk flocks have increased biosecurity and thereby will not be infected through the between subpopulation transmission route.

### Model

**Figure 1:**
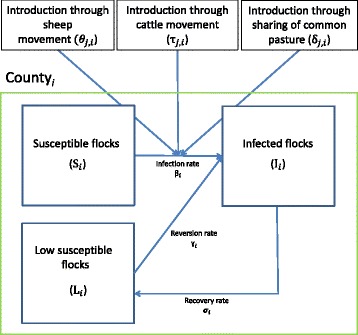
**Susceptible - Infected – Low susceptible - Infected (SILI) model of severe footrot among sheep flocks.** The simulation model was developed to estimate the potential spread of severe footrot in Norway if no elimination programme was initiated and to estimate the relative importance of the different spreading routes. The figure shows the transmission dynamics of severe footrot within one county (*i*) for one time step with possible introduction from other counties (*j*) through sheep movement, cattle movement and sharing of common pastures. The model was used to calculate all the 19 counties separately.

Equations 1–3 and Figure 1 show the differential equations of the SILI-compartmental model for one subpopulation with the possible introduction of infection from other subpopulations. The equations give the number of flocks in the susceptible (1), infected (2), and low susceptible (3) compartments in a subpopulation for each year.

1 $$ {S}_{i,y+1}={S}_{i,y}- \min \left[\left({\beta}_i\cdot {S}_{i,y}\cdot {\mathrm{I}}_{i,y}+{\displaystyle \sum_{j\ne i}}{\uptheta}_{j,\ i,\ y}+{\displaystyle \sum_{j\ne i}}{\uptau}_{j,i,\ y}+{\displaystyle \sum_{j\ne i}}{\updelta}_{j,i,\ y}\right),\ {S}_{i,y}\right] $$

2 $$ {I}_{i,y+1}={I}_{i,y}-\sigma \cdot {I}_{i,y}+ \min \left[\left({\beta}_i\cdot {\mathrm{S}}_{i,y}\cdot {I}_{i,y}+{\displaystyle \sum_{j\ne i}}{\uptheta}_{j,i,y}+{\displaystyle \sum_{j\ne i}}{\uptau}_{j,i,\ y} + {\displaystyle \sum_{j\ne i}}{\updelta}_{j,i,\ y}\right),{S}_{i,y}\right]+{\gamma}_i\cdot {\mathrm{I}}_{i,y}\cdot {L}_{i,y} $$

3 $$ {L}_{i,y+1}={L}_{i,y}+\sigma \cdot {I}_{i,y}-{\gamma}_i\cdot {\mathrm{I}}_{i,y}\cdot {\mathrm{L}}_{i,y} $$

where *i* is the subpopulation receiving the infection, *j* is the subpopulation transmitting the infection and ƴ is the time interval in years, *S* is the number of susceptible flocks, *I* is the number of infected flocks, *L* is the number of low susceptible flocks, β is the rate at which susceptible flocks become infected, σ is the rate at which infected flocks recover and hence become low susceptible flocks, γ is the rate at which low susceptible flocks become infected, θ, δ and τ is three possible ways of introduction of infection between subpopulations.

As the starting point for the simulations, the flocks infected with the disease was assigned to the infected compartment, the isolated flocks were assigned to the low susceptible compartment, and the remaining flocks were assigned to the susceptible compartment. Year was the time step and the model was run for the number of years desired.

### Adaptation of the model to footrot in Norwegian sheep flocks

A SILI-compartmental model was developed for estimating the spread of severe footrot in Norwegian sheep flocks without an elimination program. The isolated flocks in the low susceptible compartment were defined as sheep farms more than 3 km away from any other sheep farm. This was based on a study by Grøneng et al. [13] which showed that a geographic distance of more than 3 km between the main buildings of different sheep farms was not a significant risk factor in the univariable analysis. We interpret this as sheep farms with more than 3 km distance to the nearest sheep farm have a lower risk of contracting footrot.

The infection rate of footrot was calculated based on the rate of spread from the introduction of footrot in Norway in 2005 until the initiation of the elimination programme in 2009. At this time, severe footrot had only been detected in the county of Rogaland, but since different regions within the county possessed highly different rate of spread, the infection rate was expressed by a Pert distribution. Rogaland County excluding Rennesøy, Rogaland County with Rennesøy and the municipality of Rennesøy was the regions used to calculate the minimum (min), mode (mod) and maximum (max) infection rate respectively. The rates were then used in the Pert distribution.

To estimate the infection rate of the regions, the total number of sheep flocks and the number of flocks assumed to be infected with severe footrot in the region, from the introduction of the disease (2005 in Rogaland and 2006 in Rennesøy) until the initiation of the elimination programme in 2009 was used (Table 1). The infection rate was simulated based on Equations 1–3, with a constant annual recovery rate (σ) of 5.3% and a reversion rate (Ɣ) of 1/3 of the infection rate (see below for descriptions of recovery and reversion rate). The assumed number of infected flocks was the number detected in the footrot outbreak in Norway, and the predicted median number of infected flocks was as close to this number as possible. The appurtenant infection rate was used in the model. The min, mod and max predicted median number of infected flocks and the appurtenant infection rates in parentheses were 26 (1.13), 48 (1.31) and 13 (1.36), respectively (Table 1). The Pert distribution for the infection rate for Rogaland was then (β_Rog_ ~ Pert (1.13, 1.31, 1.36)).

The recovery rate was based on spread of severe footrot without an elimination programme and hence no compensation for sanitation or other measures to eliminate the disease. The recovered flocks have therefore either undergone sanitation procedure at their own cost or recovered from the disease spontaneously. Two of 38 flocks completed a successful sanitation procedure at the farmers own expense in 2008 (Vatn S, Healthy Feet project, personal communication), corresponding to a recovery rate of 5.3% per year. Some of the flocks might also recover from the disease with no intervention. Since it takes a long time for sheep in a flock to recover without human intervention [14], the percentage of these flocks is thought to be small and was not included. The recovery rate was assumed to be constant for all years.

Since none of the flocks which completed a successful sanitation procedure at the farmers own expense in 2008 was re-infected, the reversion rate could not be calculated based on data. The reversion rate was therefore sat based on knowledge of the infection dynamics. The susceptible flocks were assumed to have a three times higher infection rate than the low susceptible flocks, hence a reversion rate of (Ɣ = β/3) was used.

### Spread within subpopulations in Norway

Because of national maedi and scrapie legislation, sheep and goats are not allowed to be moved from one county to another without derogation. This gives a reduced spread from one county to another hence each of the 19 counties in Norway was assigned as a subpopulation. A SILI-compartmental model was constructed for each county. The number of sheep flocks, cattle herds and combined sheep and cattle flocks was allocated to each county from the Register of Production Subsidies of 31.07.2012 (Table 2). The register contains all holdings receiving production subsidies in Norway, hence includes >92% of the total number of sheep flocks; the ones missing are farms with very few sheep. The number was kept constant for all years.

**Table 2:** **Overview of demographic data, climatic rate and infection rate for modelling spread of severe footrot**

**County**	**N** ^**o**^ **of sheep flocks (nSh)**	**N** ^**o**^ **of cattle herds (nCa)**	**N** ^**o**^ **of combined flocks (** ***nShCa***)	**N** ^**o**^ **of isolated flocks**	**Mean number of neighbouring flocks (** ${\overline{n}}_{1\boldsymbol{km},\boldsymbol{i}}$ **)**	**Climatic rate (Cl**_**i**_ **/Cl**_**Rog**_ **)**	**Infection rate** **(β) (minimum, mode, maximum)**
Østfold	160	360	43	49	0.2	0.61	0.04, 0.05, 0.05
Akershus	226	359	45	43	0.8	0.61	0.17, 0.19, 0.20
Oslo	8	5	4	6	0.0001*	0.67	0.01, 0.01, 0.01
Hedmark	669	1108	200	99	1.4	0.51	0.24, 0.28, 0.29
Oppland	1323	2224	324	60	1.6	0.51	0.28, 0.32, 0.34
Buskerud	552	593	111	53	1.5	0.56	0.29, 0.33, 0.35
Vestfold	129	225	29	25	0.3	0.69	0.07, 0.08, 0.09
Telemark	372	360	72	54	1.3	0.67	0.30, 0.35, 0.36
Aust-Agder	220	250	47	60	0.8	0.76	0.21, 0.24, 0.25
Vest-Agder	450	583	150	45	1.2	0.93	0.38, 0.44, 0.46
Rogaland	2597	2735	1297	20	3.3	1	1.13, 1.31, 1.36
Hordaland	1997	1457	603	37	2.3	1	0.79, 0.91, 0.95
Sogn og Fjordane	1617	1756	625	59	2.5	0.97	0.83, 0.96, 1.00
Møre og Romsdal	1053	1880	358	94	1.2	0.89	0.37, 0.42, 0.44
Sør-Trøndelag	728	1729	187	99	1.1	0.62	0.23, 0.27, 0.28
Nord-Trøndelag	524	1737	178	98	0.6	0.67	0.14, 0.16, 0.17
Nordland	1045	1366	272	169	0.8	0.73	0.20, 0.23, 0.24
Troms	522	442	92	92	0.8	0.5	0.14, 0.16, 0.16
Finnmark	123	192	26	42	0.5	0.38	0.07, 0.08, 0.08

*In the county of Oslo no flocks had any neighbouring flocks within 1 km, hence the value was set at 0.0001.

The data are displayed for all the 19 counties in Norway. The number of sheep and cattle flocks includes the number of combined flocks. The number of isolated flocks (no other flocks within 3 km), the mean number of neighbouring flocks (sheep farms within a distance of 1 km), and the climatic rate (Cl_*i*_ **/**Cl_Rog_ **)** was calculated on the basis of the geographical co-ordinates of the building of the sheep farms. The infection rate (β) was the rate of susceptible flocks becoming infected due to within county transmission.

The infection rate calculated earlier was only based on the rate of spread within the county of Rogaland. To calculate the rate of spread within each of the other counties, values which would interfere with the spread of footrot are quantified and used to adjust the minimum, mode and maximum within county infection rate for Rogaland.

One of the values expected to interfere with the infection rate of footrot was the climate within each county as this is an important factor for the survival of *D. nodosus* and for the initiation and development of ovine footrot [1,15]. In particular the precipitation and temperature are considered important for the spread of footrot [15,16]. The geo-coordinates of all sheep farm buildings in Norway (f) (the Agricultural Property Register, 2011) were linked to a mean value of precipitation $\left({\overline{p}}_{f,\ i}\right)$ and temperature (${\overline{t}}_{f,\ i}\Big)$ from May until October (Norwegian Meteorological Institute, data from 1971 till 2000). By summarising the mean daily precipitation in mm and mean daily temperature in degrees Celsius of the individual sheep farms in a county, and dividing by the number of sheep farms in that county (*N*_*f,i*_), a climatic value (called *Cl*_*i*_) was calculated for each of the 19 counties (Equation 4).

4 $$ C{l}_i=\frac{{\displaystyle {\sum}_{f\kern0.5em }}{\overline{p}}_{f,\ i} + {\displaystyle {\sum}_f}\kern0.5em {\overline{t}}_{f,\ i}}{N_{f,\ i}} $$

The fraction between the climatic factors in county *i* and the climatic factor in Rogaland was incorporated in Equation 5 to adjust the infection rate within each county.

Another value expected to interfere with the infection rate of footrot was the density of sheep farms within each county. Grøneng et al. [13] showed that a risk factor for contracting the disease is a sheep farm located less than 1 km from a sheep farm positive for severe footrot. The distances between farms were calculated based on the locations of the main building on each farm. Hence, for each sheep farm, the number of other sheep farms within 1 km (neighbour farms) was obtained. Based on this, the mean number of neighbour farms to the sheep farms within each county (${\overline{n}}_{1 km,i}$) was calculated (Table 2). The fraction between the mean number of sheep farms within 1 km in county *i* and the county Rogaland was used to adjust the infection rate in county *i* (Equation 5). By using the knowledge of the spread of disease in the county of Aust-Agder, the effect of the fraction between counties was adjusted. In 2013, 14 flocks in the county of Aust-Agder were diagnosed with severe footrot, and epidemiological investigations indicate that sheep moved from the county of Rogaland in 2006 were the source. The spread from the introduction in 2006 to 2013 was simulated based on Equations 1–3, and a value k, adjusting the effect of the density factor between Aust-Agder and Rogaland, was chosen so that the median value of 2000 replicates matched the number of infected flocks in Aust-Agder in 2013. A median of 14 (range 1–26) infected flocks was predicted for *k* = 2.3 (Equation 5).

For all counties except Rogaland, a county specific min, mod and max infection rate was estimated by adjusting the min, mod and max infection rate for Rogaland with the constant k, the mean number of sheep farms within 1 km and climatic value for the respective counties. These values were then used in a Pert distribution, where a new value was estimated for each county and each replicate (Equation 5).

5 $$ \upbeta \mathrm{i}\sim \mathrm{Pert}\ \left({\beta}_{Min,Rog}\cdot \sqrt[2.3]{\frac{{\overline{n}}_{1 km,i}\ }{{\overline{n}}_{1 km,Rog}\ }}\cdot \frac{C{l}_i}{C{l}_{Rog}},\ {\beta}_{Mod,Rog}\cdot \sqrt[2.3]{\frac{{\overline{n}}_{1 km,i}\ }{{\overline{n}}_{1 km,Rog}\ }}\cdot \frac{C{l}_i}{C{l}_{Rog}},\ {\beta}_{Max,Rog}\cdot \sqrt[2.3]{\frac{{\overline{n}}_{1 km,i}\ }{{\overline{n}}_{1 km,Rog}\ }}\cdot \frac{C{l}_i}{C{l}_{Rog}}\right) $$

where *i* is the county, and *Rog* is Rogaland County.

The recovery rate was not expected to differ between counties and was expected to be constant for every year. The reversion rate for each county was defined as one third of the infection rate (Ɣ_*i*_ = β_i_/3).

### Spread between subpopulations in Norway

The spread of footrot between counties in Norway was modelled taking three potential transmission routes into consideration: 1) movement of sheep between counties, 2) movement of cattle between counties, and 3) introduction by sharing of common pastures (Figure 1).

#### *Introduction from other counties through sheep movement* (*θ*)

Although there is a general ban on movement of sheep from one county to another because of maedi and scrapie, derogations from the legislation can be authorised by the Norwegian Food Safety Authority. Two movements of sheep between counties were recorded in 2013 (MATS, the supervision system of the Norwegian Food Safety Authority). There may have been movements of sheep that have not been reported to the central Food Safety Authority, but these are believed to be minimal. We therefore assumed that some of the sheep in 0.05% of the flocks in a county would be moved to each of the neighbouring counties each year. In addition, some of the sheep in 0.025% of the flocks in a county would move sheep to each of the counties bordering on neighbouring counties each year. Thus the number of movements from county *j* to county *i* was estimated (*MSh*_*j,i*_), and used to calculate the introduction of severe footrot to other counties (Equation 7). For Norway as a whole, this is equivalent to approximately 44 between county movements of sheep each year. As this is more than reported to the Norwegian Food Safety Authority, we believe the effect of moving sheep across county borders has been overestimated rather than underestimated.

Only movement of sheep that is infected with footrot can transmit the disease to other sheep flocks. This depends on the probability that sheep from an infected flock are moved $\left(\frac{I_j}{nS{h}_j} \cdot MS{h}_{j,i}\right)$, and also on the probability that at least one of the sheep moved is infected (*ProbMove*). The *ProbMove* is based on the number of sheep moved and the prevalence of infected sheep within the flock. The minimum *ProbMove* value was based on movement of one sheep from a flock with an infection prevalence of 0.01. The maximum *ProbMove* value was based on movement of five sheep from a flock with a prevalence of 0.65. The values were calculated to be 0.01 and 0.995 as shown in Equation 6. Consequently, a uniform distribution with a minimum and maximum value of the *ProbMove* was used (ProbMove ~ Unif (0.01, 0.995)) in Equation 7.

6 $$ MaxProbMove=1 - {\left(1- Prevalence\right)}^{NumberMoveSheep} $$

The number of sheep moved was based on the knowledge that mostly rams are purchased, and since the sheep flocks are small, rarely more than two rams are acquired at the same time. The lowest prevalence was based on one infected sheep in a flock of 100 sheep. The highest prevalence was based on PCR examination of all sheep in three flocks infected with severe footrot, and the median of these values was used. This was chosen since only sheep from flocks with a veterinary health certificate may be moved across county borders. We therefore believe that flocks with a prevalence above 0.65 would not be allowed to move sheep because they would show pronounced clinical signs of footrot.

The introduction of severe footrot from other counties by sheep movement is shown in Equation 7, where the percentage of susceptible sheep flocks in county *i*$\left(\frac{S_i}{nS{h}_i}\right)$. was included in order to calculate the probability of an infected sheep arriving at a susceptible sheep flock. We expect that a sheep which is infected with footrot would infect a flock of susceptible animals.

7 $$ {\theta}_{j,i}=\frac{I_j}{nS{h}_j} \cdot MS{h}_{j,i} \cdot ProbMove \cdot \frac{S_i}{nS{h}_i} $$

where *i* is the county receiving infectn, and *j* is the county transmitting the infection, *I* is the number of infected sheep flocks, *nSh* is the total number of sheep flocks, *MSh* is the number of flocks that have moved sheep, and *S* is the number of susceptible sheep flocks.

#### *Introduction from other counties through cattle movement* (τ)

Cattle that have been in contact with infected sheep may be carriers of virulent *D. nodosus* and transmit the infection to sheep [17,18]. Hence, virulent *D. nodosus* might be introduced to a new county by movement of cattle. The number of moved cattle aged >1 year (*MCa*) in 2007 was retrieved from the Norwegian National Cattle Register (Norwegian Food Safety Authority) (Table 2). Cattle aged <1 year were not included as calves are usually not in contact with sheep, and the probability of a calf being infected by its mother and remain infected until moved to another flock was expected to be minimal. Only information from 2007 was available. In cases where there was no registered movement between neighbouring counties in 2007, movement of one head of cattle was imputed. The register also included records of movement of cattle without information about which county they were moved from. These were included by giving the unknown county the mean number of cattle flocks, the mean number of combined flocks and the mean number infected for all the counties. The number of infected sheep flocks in a county that transmit the disease (*I*_*j*_), the number of sheep flocks in the county *j* (*nSh*_*j*_), the number of cattle flocks in counties *i* and *j* (*nCa*_*i*_*, nCa*_*j*_), and the number of combined cattle and sheep flocks in counties *i* and *j* (*nShCa*_*j*_, *nShCa*_*i*_) (Table 2) are used to calculate the probability of severe footrot being introduced from other counties by movement of cattle. The probability of a sheep infecting cattle (Sh2Ca) and vice versa (Ca2Sh) was also needed for the calculation. On the basis of a study by Knappe-Poindecker et al. [18], the value was found to be 0.1 (gelatin gel test showed five of fifty cattle to be positive after co-grazing with sheep), while a study by Rogdo et al. [19] found this probability to be 0.3 (18 of 58 cattle were PCR-positive for footrot with serogroup A). The probability of sheep infecting cattle and vice versa was given by a uniform distribution (Ca2Sh ~ *Unif *(0.1, 0.3), *Sh*2*Ca* ~ *Unif* (0.1, 0.3)), a new value was generated for each movement. The percentage of susceptible sheep flocks in county *i*$\left(\frac{S_i}{nS{h}_i}\right)$ was included to enable calculation of the probability of infected cattle entering a susceptible sheep flock. Equation 8 expresses the introduction of severe footrot from other counties through movement of cattle:

8 $$ {\uptau}_{j,i}={I}_j\cdot \frac{nShC{a}_j}{nS{h}_j}\cdot Sh2Ca\cdot \frac{MC{a}_{j,i}}{nC{a}_j}\cdot \frac{nShC{a}_i}{nC{a}_i}\cdot Ca2Sh \cdot \frac{S_i}{nS{h}_i} $$

where *i* is the county receiving infection, and *j* is the county transmitting the infection.

#### *Introduction from other counties through sharing of common pasture (*$\delta$)

In Norway, many sheep flocks are transported to common pastures during the summer. This is mainly pastures situated in mountain areas. This is an old tradition, and it is important both for reducing the farmer’s feed expenses and for conserving the countryside. There are nearly 1000 common pasture groups in Norway, each with several members and a designated area for their sheep to graze (Norwegian Forest And Landscape Institute). The organisation of the pasture groups is quite complex, with some common pasture areas crossing county borders. Some pasture groups also have members from several counties. Information about common pastures that share borders with common pastures in other counties and common pastures that have members from different counties are included in the estimation of cross-county transmission on pasture. In these pastures there are no fences or other barriers, with the result that sheep from different counties can mix and transfer infection. The spread of severe footrot on common pasture was calculated in a same way as the within county infection rates (Equation 5) by adjusting the infection rate for Rogaland for differences in sheep flock density and climate. Since sheep flocks are free ranging on common pasture, it is difficult to estimate the mean number of flocks within 1 km, as was done when calculating the within county infection rates. But sheep flocks are often put on common pasture at different times and in different areas, and 1–2 ewes with their lambs tend to keep together within a small area and rarely be in contact with other sheep. Assuming maximum dispersion of flocks on common pasture, we calculated the mean number of flocks per 1 km^2^ for each of the common pastures and used this as a proxy for the number for flocks within 1 km of each other. The higher the density of sheep flocks on common pasture, the higher the infection rate then will be. The mean number of flocks within 1 km^2^ on the common pastures where flocks from county *j* and *i* can be in contact with each other (${\overline{N}}_{1k{m}^2, past,j,i}$) was calculated as shown in Equation 9 by adding the density of common pastures in county j and *i* which have a common border (*Bpast*) to the density of common pasture which have members from both counties *j* and *i* (*Mpast*). This was used in Equation 10 to calculate the common pasture infection rates.

9 $$ {\overline{N}}_{1k{m}^2, past,j,i}=\frac{{\displaystyle {\sum}_{Bpast}}\left(\frac{\left({N}_{f(j)}/{A}_{past(j)} + {N}_{f(i)}/{A}_{past(i)}\right)}{2}\right) + {\displaystyle {\sum}_{Mpast}}\left({N}_{f,j,i}/{A}_{past\left(j,i\right)}\right)}{n_{Bpast} + {n}_{Mpast}} $$

where *N*_*f*_ is the number of sheep flocks on pasture, *A*_*past*_ is the geographic area of the pasture in km^2^ and *n* is the number of pastures.

The climate of the common pastures (*Cl*_*past*_) could not be calculated in the same way as the within county climate because we did not have specific geographical points, but rather large areas across which the sheep flocks were spread. The common pastures are often situated at a higher altitude than the general location of sheep farms, and the climate is often colder and dryer. Given this knowledge, we believe that the climate on common pasture has a lower value than the climate in any of the counties, so the climatic rate of common pasture (*Cl*_*past*_*/Cl*_*Rog*_) was assumed to be 0.3, lower than the lowest climate rate (Table 2). The climatic rate was constant for all years, and was used in the calculation of the common pasture infection rates as shown in Equation 10.

The common pasture infection rate was calculated in the same way as the within county infection rates (Equation 5) with a Pert distribution for each county and each iteration (Equation 10).

10 $$ {\beta}_{past,j,\ i} \sim \mathrm{Pert}\ \left({\beta}_{Min,Rog}\cdot \sqrt[2.3]{\frac{{\overline{N}}_{1k{m}^2, past,j,i}}{{\overline{n}}_{1 km,Rog}\ }}\cdot 0.3,\ {\beta}_{Mod,Rog}\cdot \sqrt[2.3]{\frac{{\overline{n}}_{1k{m}^2, past,j,i}}{{\overline{n}}_{1 km,Rog}\ }}\cdot 0.3,\ {\beta}_{Max,Rog}\cdot \sqrt[2.3]{\frac{{\overline{n}}_{1k{m}^2, past,j,i}}{{\overline{n}}_{1 km,Rog}\ }}\cdot 0.3\right) $$

The introduction of severe footrot from other counties through sharing of common pasture was calculated on the basis of the common pasture infection rate (*β*_*past,j,i*_), the percentage of infected flocks in county *j*$\left(\frac{I_j}{nS{h}_j}\right)$, and the percentage of susceptible flocks in county *i*$\left(\frac{S_i}{nS{h}_i}\right)$. The number of flocks from county *i* (*n*_*past,i*_) and county *j* (*n*_*past,j*_) on common pasture was also included to calculate the number of flocks in county *i* that were newly infected by sharing common pasture with county *j* (Equation 11).

11 $$ {\updelta}_{j,\ i} = {\upbeta}_{\mathrm{past},\ \mathrm{j},\ \mathrm{i}} \cdot \frac{I_j}{nS{h}_j}\cdot {n}_{past,\ j} \cdot \frac{n_{past,\ i}}{\left({n}_{past,\ j} + {n}_{past,\ i}\right)} \cdot \frac{S_i}{nS{h}_i} $$

where *i* is the county receiving infection, and *j* is the county transmitting the infection.

### Model for Norway

As the starting point for the simulations, one flock in the county of Rogaland was assigned to the infected compartment, the isolated flocks in each county were assigned to the low susceptible compartment in the respective counties, and the remaining flocks in each county were assigned to the susceptible compartments. When the probability of transferring flocks between compartments resulted in decimal number of flocks, the decimal number was converted to an integer by performing a Bernoulli trial with the decimal fraction as the probability. The county results were aggregated to give the results for Norway.

### Scenarios

#### Basic scenario

The basic scenario was simulation of the spread of severe footrot without any elimination or control with input values as presented in Tables 1 and 2. For all the scenarios where input parameters were changed, the basic scenario was used as the reference.

#### Scenarios with different control measures

The disease can be controlled by reducing the within county or between county transmissions compared to the basic scenario. Scenarios with a 20%, 40%, 60% and 80% lower infection rate within the counties were modelled. Scenarios with 20%, 40%, 60%, 80% and 100% less movement of sheep between counties were modelled. Scenarios with 20%, 40%, 60%, 80% and 100% less movement of cattle between counties were modelled. Scenarios with 20%, 40%, 60%, 80% and 100% fewer flocks sharing common pasture were modelled.

#### Scenarios with increased between county transmission

Increased between county movement of both sheep and cattle, and an increased number of flocks on common pastures are scenarios that we might see in the future. Hence the importance of this factor is highlighted. A five-fold and ten-fold increase was modelled.

### Sensitivity analyses

By increasing and decreasing the basic scenario parameters one by one, an indication of the robustness of the model and the sensitivity of the model parameters is found. The sensitivity analysis was performed by stepwise increasing and decreasing of the parameters, starting with 80%, then 60%, 40% and 20%. The analysis was continued until the number of infected flocks did not deviate by more than 5% compared to the basic model. Thus, only the 80% increase and decrease was performed for the parameters which showed little variance in the results compared to the basic scenario. The parameters included in the sensitivity analysis were the infection rate, recovery rate, reversion rate, climatic value, climatic rate on common pasture, number of farms within 1 km (neighbouring flocks) and number of farms within 3 km (isolated flocks).

### Model simulations

The model was run from 2005 and 30 years onward. In addition, the basic scenario where the time interval was extended to the year 2100 was made. The intention was to capture the percentage of flocks in each of the compartments when the equilibrium state was reached. The model was run using R v2.15.1 [20] and the additional package deSolve [21]. For each simulation of a scenario, 2000 replicates were made.

## Results

### Basic scenario

**Figure 2:**
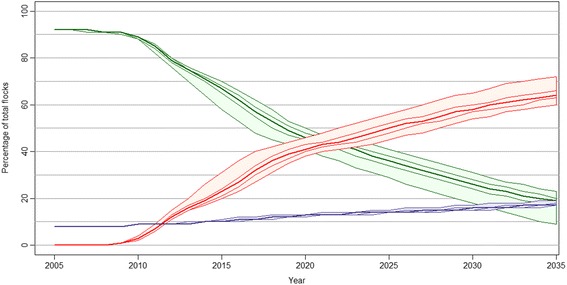
**Simulated development of severe footrot in Norwegian sheep flocks without an elimination programme.** The percentage of susceptible (green), infected (red) and low susceptible (purple) flocks are shown for each of the years 2005 – 2035 with the median value and 2.5, 25, 75 and 97.5 percentiles.

In the basic scenario, severe footrot was estimated to have spread to six of the 19 counties and 16% of the sheep flocks in Norway by 2013, and 16 counties and 64% of the flocks were infected by 2035 (Figure 2). In 2100, severe footrot was estimated to be spread to all counties except Oslo, and to 76% of the sheep flocks.

**Figure 3:**
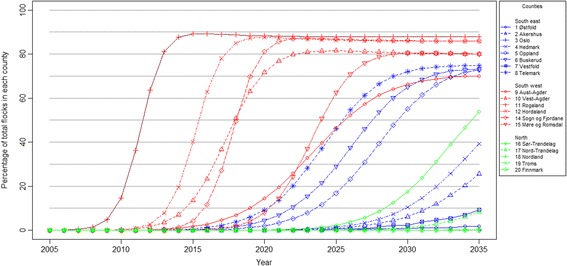
**Simulated spread of severe footrot without an elimination programme in the 19 Norwegian counties.** The median percentage of infected flocks within each county for each year in the period 2005 – 2035.

**Figure 4:**
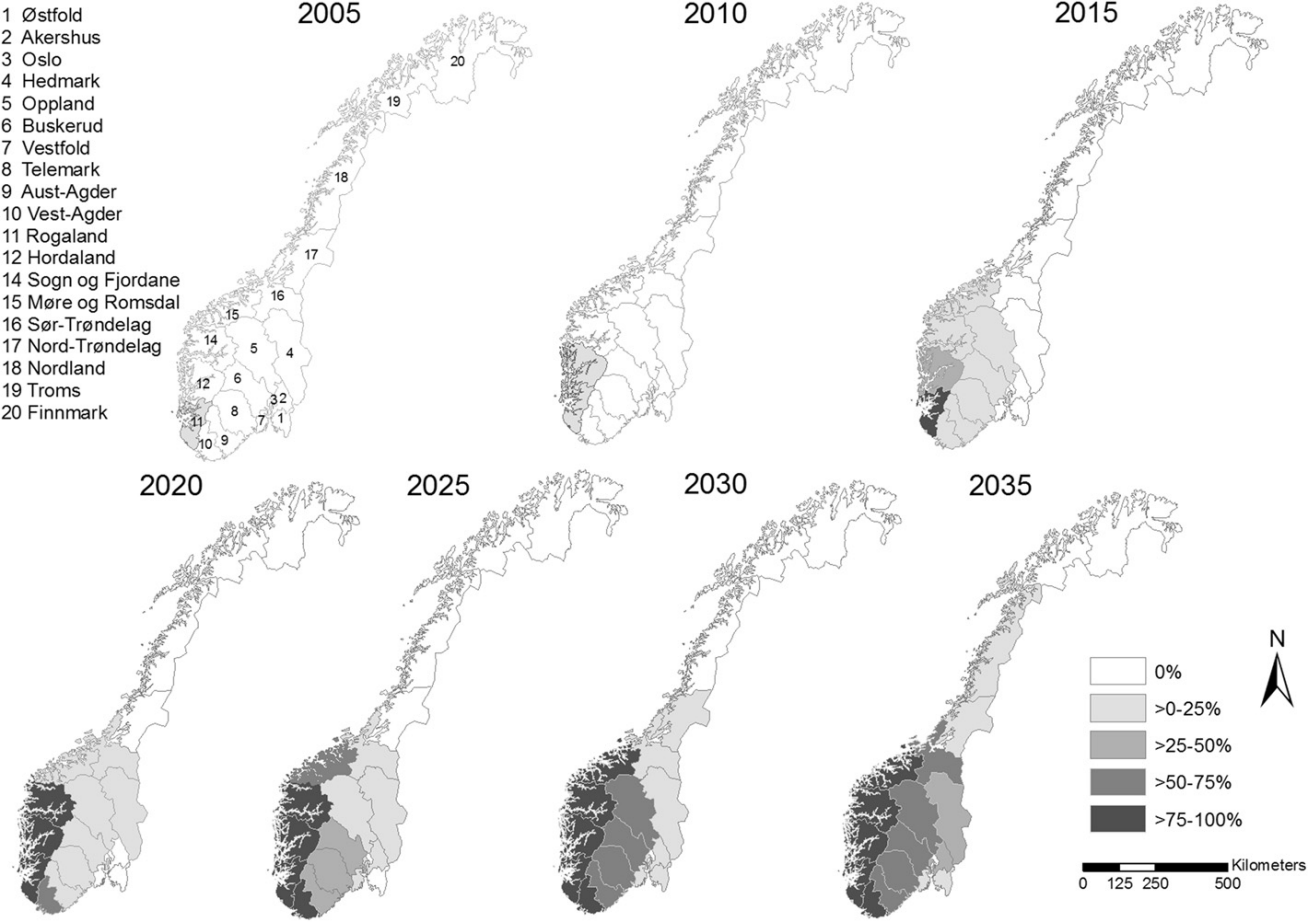
**Simulated geographical spread of severe footrot in Norwegian sheep flocks without an elimination programme.** The maps show the spread of severe footrot in the 19 counties of Norway at five-year intervals. The intensity of the grey shading shows the percentage of infected flocks in each of the counties.

The counties in the south-west of Norway were infected during the early years; after that, the remaining counties in southern Norway were infected, except Oslo, where none of the eight flocks were infected by 2035. Nor did the two northernmost counties, i.e. Troms and Finnmark, experience an introduction of severe footrot during the simulated period (Figures 3 and 4).

After the initial introduction of infection into a county, the model estimates that it takes four to twenty years before approximately 5% of the sheep flocks in the county are infected. Thereafter, one to nineteen years elapses before more than 30% of the sheep flocks in the county are infected. The steepest increase in the number of infected sheep flocks is observed in the five counties Rogaland, Hordaland, Vest-Agder, Sogn og Fjordane and Møre og Romsdal. Infected flocks in these counties increase from 5% to 60% within two to five years, while in the other counties it takes from eight to more than twenty years to reach this percentage (Figure 3).The counties of Rogaland and Finnmark have the highest and lowest percentages of infected flocks in 2100, at 88% and 7%, respectively, when the county of Oslo is excluded (0%). A state of equilibrium between the compartments are reached in the year 2068, when the number of infected flocks and low susceptible flocks stabilises at 76% and 24%, respectively.

#### Scenarios with different control measures

**Figure 5:**
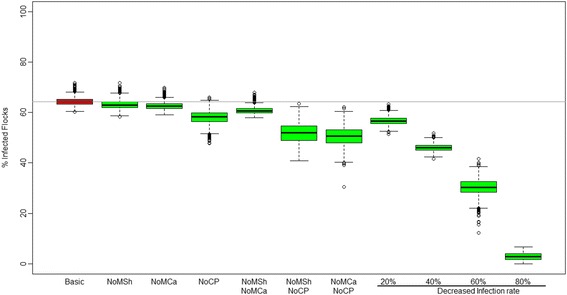
**Estimated percentage of infected flocks in 2035 using different strategies for reducing severe footrot.** The box-and-whiskers plot shows the distribution of the percentage of total flocks infected in Norway in 2035 based on 2000 replicates. The basic scenario is shown in red and various reduction strategies of the infection in green. The box represents the 25 and 75 percentiles and the black line inside the box represents the median value. Circles outside the whiskers are outliers. BasicS = Basic Scenario, NoMSh = exclusion of sheep movement, NoMCa = exclusion of cattle movement, NoCP = exclusion of common pasture, 20%, 40%, 60% and 80%: decrease in infection rate by 20%, 40%, 60% and 80%, respectively.

A 20% and 40% reduction in the infection rate results in 57% and 46% infected flocks respectively in 2035 (Figure 5). The exclusion of one of the between counties transmission routes at a time, while keeping the other two routes, reduces the number of infected flocks (Figure 5). The prohibition of common pasture reduces the number of infected flocks in 2035 by 9% compared to the basic scenario. The prohibition of movement of cattle reduces the number of infected flocks by 2%, and delays the introduction of severe footrot to one more county compared to the basic scenario, i.e. the county of Østfold is not infected by 2035 in this scenario. By concurrent exclusion of two between county transmission routes, the number of infected flocks is further reduced compared to the basic scenario. Excluding both the movement of cattle and common pasture results in a 20% decrease in the number of infected flocks in 2035 compared with the basic scenario.

#### Scenarios with increased between county transmission

A ten-fold increase in the movement of sheep, the movement of cattle, and the use of common pasture increase the number of infected flocks in 2035 by 3%, 9% and 17%, respectively. The spread of severe footrot is further extended (compared to the basic scenario) to the county of Troms in the scenarios with increased movement of cattle and increased use of common pasture, and also to the county of Oslo in the scenario with increased movement of cattle.

#### Sensitivity analysis

**Figure 6:**
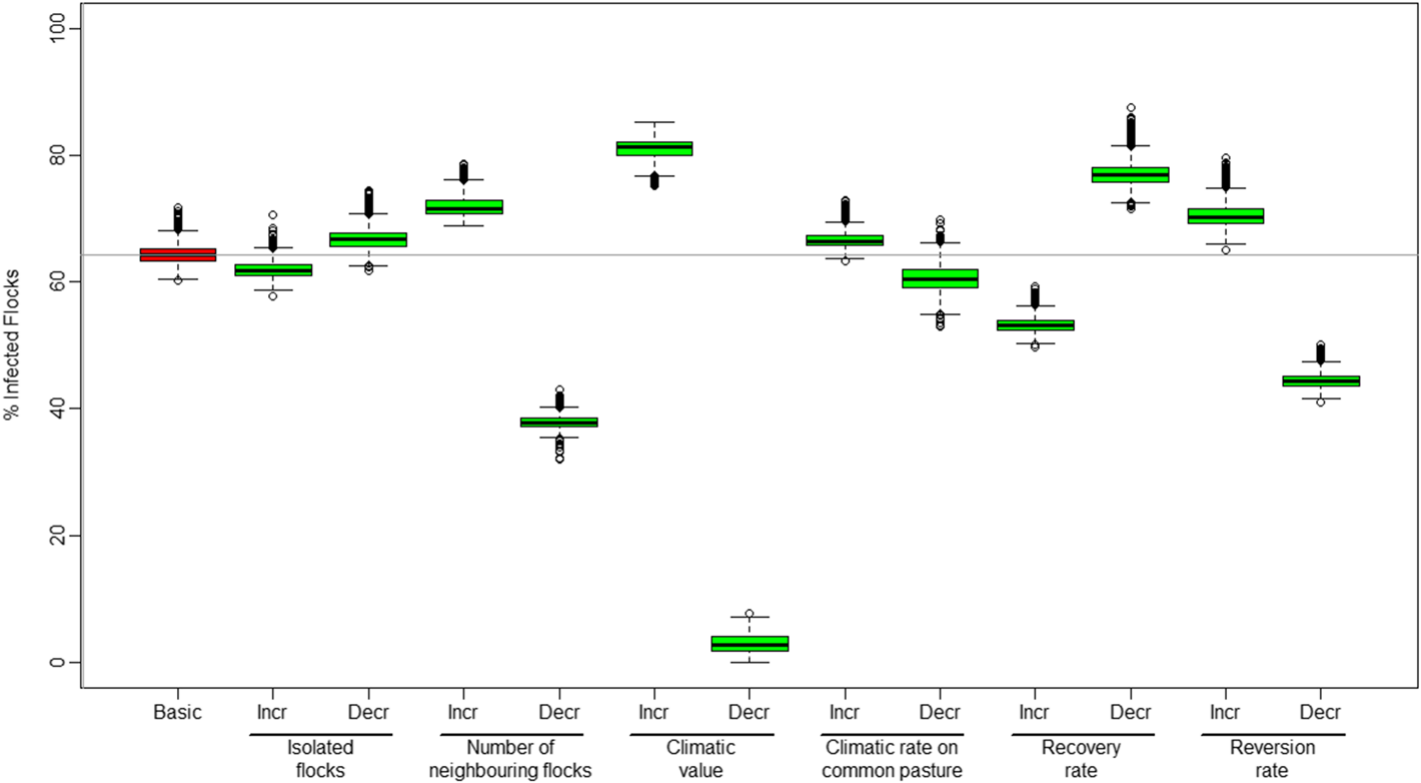
**Sensitivity analysis of parameters used in a model for the spread of severe footrot.** The box-and-whiskers plot shows the distribution of the estimated percentage of flocks infected with severe footrot in Norway in 2035 based on 2000 replicates. The basic scenario is shown in red and the 80% increase and decrease in the variables in the model is shown in green. The box represents the 25 and 75 percentiles and the black line inside the box represents the median value. Circles outside the whiskers are outliers. BasicS = Basic scenario, Incr = 80% increase in the variable and Decr = 80% decrease in the variable.

In the sensitivity analysis, the variables with a more than 5% decrease or increase in the number of infected flocks compared to the basic scenario in 2035 are listed underneath. When reducing the climatic value with 80, 60, 40 and 20%, the number of infected flocks was reduced to 3%, 30%, 44% and 55%, respectively. When increasing the climatic value with 80, 60, 40 and 20%, the number of infected flocks was increased to 81%, 78%, 73% and 70%, respectively. When reducing the number of neighbouring flocks with 80, 60 and 40%, the number of infected flocks was reduced to 38%, 48% and 55%, respectively. When increasing the number of neighbouring flocks with 80, 60 and 40%, the number of infected flocks was increased to 72%, 70% and 69%, respectively. When reducing the reversion rate by 80, 60 and 40%, the number of infected flocks was reduced to 44%, 53% and 58%, respectively. When increasing the reversion rate with 80, 60, 40 and 20%, the number of infected flocks was increased to 70%, 69% and 68%, respectively. When reducing the recovery rate by 80, 60 and 40%, the number of infected flocks was increased to 77%, 74% and 70%, respectively. When increasing the recovery rate by 80, 60 and 40%, the number of infected flocks was reduced to 53%, 56% and 59%, respectively. The variables which with an 80% increase or decrease deviated 5% or less from the basic scenario were; increased and decreased 3 km distance and increased and decreased climate on common pasture (Figure 6).

## Discussion

A simulation model is a useful tool to predict the spread of disease in a population. But spread of an infectious disease is a complex phenomenon with many interacting factors. Hence the development of a model must be based on knowledge of the specific disease in question and the routes of spread within the population as the model assumptions and the input variables used are important for obtaining a reliable result.

Most of the assumptions and input variables in this study have been based on observed parameters of the population, management and climate in Norway. In addition, the infection rate was based on observed values of the spread of footrot in Rogaland from introduction in 2005 until implementing the elimination program in 2009, and then adjusted to the other counties in Norway by using the observed spread in Aust-Agder. Data on spread for more than four years and more than two counties would have been desirable, but since an elimination program was implemented in Norway, such data was not available.

The sensitivity of the input parameters has also been examined. The variables found important in the sensitivity analysis were changes in the climatic value, the number of neighbouring flocks, the recovery, and the reversion rate. As expected, the climatic value is an important parameter for the spread of footrot. The values used in the model were based on observed mean precipitation and temperature for a 30 year period. More than a 20% increase or reduction of the observed value is not expected. Hence up to 14% decrease or 9% increase in the number of infected flocks compared to the basic scenario might be possible. The number of neighbouring flocks is also an important parameter. This value was based on the geographical coordinates of sheep farms. A higher or lower density of sheep farms might be possible, but we do not expect more than 20% change in this factor during the modelled 30 years. This would result in less than 5% deviation compared to the basic model. The recovery rate was based on data from the Healthy Feet project, and an increase or decrease in this value of more than 20% is not expected. With an increase/decrease of 20% the deviation from the basic scenario was less than 5%. The reversion rate is an important model parameter, and data on this value would have been desirable, but this is one of the few parameters in the model for which no data is available. A 40% change in this variable gives a more than 5% deviation in the result compared to the basic scenario. We cannot exclude the possibility that the reversion rate can have other values than the ones modelled. Low reversion rate is an advantage, and this can be achieved by good biosecurity measures. The rest of the variables did not change the outcome in the sensitivity analysis.

The reliability of the model can be assessed by comparing the infection dynamics in the model with what is expected based on knowledge of the disease and the spread in other populations.

Severe footrot was not introduced into the two northernmost counties in Norway in the period covered by the simulation. These counties are situated far from the county of Rogaland and to reach them the infection would have to cross several county borders that act as barriers for the transmission of the infection. In addition, the average temperature decreases going north in the country, resulting in a climate that is less favourable to the spread of footrot. A delayed introduction and spread to these counties, as predicted by the model, would accordingly be expected.

Another of the factors which increase the reliability of the model is the fact that the steepest increase in number of infected sheep flocks was seen in five counties that are all characterized by having a wet, warm climate and a relatively high density of sheep flocks compared to the other counties in Norway. These factors are known to enhance the development and spread of *D. nodosus*, resulting in a high within county infection rate, hence a steep increase is expected.

We also compared the modeled results with parameters from the UK, where footrot is endemic. In a study, 86% of the sheep farmers in the UK reported to have footrot within a twelve-month period, and more than 95% had experienced footrot at some time [22]. This is similar to the situation in Rogaland county which stabilised at 88%. The overall prevalence of infected flocks in Norway stabilised at a lower level, but this was as expected since the other counties with the exception of Hordaland, have a climate less favourable for footrot (Table 2).

Even though this model was based on the factors specific for Norway, a similar approach can be used to predict the spread of disease in other populations by estimating the input variables specific to the disease and the country or region in question.

### Basic scenario

Extensive spread of severe footrot, in terms of both the number of infected flocks and the number of counties affected is predicted within 30 years (Figures 2, 3 and 4). This results in a large proportion of the Norwegian sheep population being affected by pain, lameness and welfare problems which would have a high economic cost for the sheep industry [23]. A comparison of the predicted number of infected flocks with the cumulative number of flocks diagnosed with severe footrot in 2013 appears to show that the footrot elimination programme initiated in 2009 was highly effective. This shows the importance of early implementation of an elimination programme for a newly introduced disease like severe footrot.

### Scenarios with different control measures

The most effective way to reduce the spread of severe footrot was by decreasing the within county infection rate (Figure 5). This could be achieved by reducing contact between flocks or by reducing the environmental load of virulent *D. nodosus,* for example by footbathing, culling diseased sheep or eliminating severe footrot from the flock. In the event of an extensive outbreak of severe footrot, we believe that some farmers would implement control measures to reduce the welfare problem in their flock. A 20% or possibly a 40% decrease in the infection rate might be realistic, which would decrease the number of infected flocks in 2035 by 11% and 28%, respectively, compared with the basic scenario where no control measures are included.

The exclusion of one of the between counties transmission routes at a time, keeping the other two routes in the model, resulted in only a small decrease in the number of infected flocks in 2035. When two of the three transmission routes were excluded, a larger reduction in the number of infected flocks in 2035 was seen. When sheep movement was the only between county transmission route, the number of infected flocks decreased by 20%. Of the between county transmissions, this was the scenario with the largest deviation from the basic scenario. The low number of sheep moved across county borders is the main reason why the spread of disease is slowest for this route. National maedi and scrapie legislation prohibits the movement of sheep across county borders without derogation. This shows that keeping this transmission route only in the model at the current level limits the spread. This reflects the importance of the legislation in decreasing the spread of disease across county borders. With no such legislations, more sheep would be moved across the county borders and the spread of sheep diseases to other counties would be faster. The sheep industry in Norway supports the derogations for moving sheep across county borders, hence an increase is not expected.

### Scenarios with increased between county transmission

Increased use of common pasture and movement of cattle gave the highest increase in the number of infected flocks of the between county transmissions. This shows the importance of the risk of spreading severe footrot by these means. But an extensive increase in these routes of transmissions is not expected as they are not restricted with legislations, and therefore not a major concern for the control of footrot in Norway.

The county-specific infection rates were based on the spread of severe footrot in Rogaland and adjusted to other counties by taking account of differences in climate and sheep density. The adjustment factors were fitted to the spread in Aust-Agder. We cannot exclude the possibility that other ways of generating the correction factors would be better. In view of the importance of the model results, data to validate the adjustment factors would have been beneficial, but such data does not exist for Norway.

In conclusion, a simulation model is a useful tool to estimate the spread of an infectious disease, but care must be taken so that model assumptions and values used are reasonable as the results are highly dependent on these. By using sensitivity analysis and assessing the consistency with spread in other populations, the reliability of the model can be assessed.

The spread of severe footrot in Norway without an elimination programme would have been extensive. Control measures decreasing the within county infection rate would delay the spread, but a ban on a single of the between county infection routes would not reduce the spread substantially. This shows the large effect, and the importance of initiating an elimination programme to prevent a large proportion of the Norwegian sheep population from being faced with pain, lameness and welfare problems. We cannot exclude the possibility of disease being introduced and spread by other means than those modelled, but we do believe that the model predicts a possible scenario for how the disease would develop in Norway without an elimination programme.
